# Studies of the Anti-amnesic Effects and Mechanisms of Single and Combined Use of Donepezil and Ginkgo Ketoester Tablet on Scopolamine-Induced Memory Impairment in Mice

**DOI:** 10.1155/2019/8636835

**Published:** 2019-02-18

**Authors:** Jing Zhang, Jun Wang, Gui-Sheng Zhou, Ya-Jie Tan, Hui-Juan Tao, Jia-Qian Chen, Zong-Jin Pu, Jia-Yan Ma, Wen She, An Kang, Yue Zhu, Pei Liu, Zhen-Hua Zhu, Xu-Qin Shi, Yu-Ping Tang, Jin-Ao Duan

**Affiliations:** ^1^Jiangsu Collaborative Innovation Center of Chinese Medicinal Resources Industrialization, and Jiangsu Key Laboratory for High Technology Research of TCM Formulae, and National and Local Collaborative Engineering Center of Chinese Medicinal Resources Industrialization and Formulae Innovative Medicine, Nanjing University of Chinese Medicine, Nanjing 210023, Jiangsu Province, China; ^2^Key Laboratory of Shaanxi Administration of Traditional Chinese Medicine for TCM Compatibility, and Shaanxi Key Laboratory of Chinese Medicine Fundamentals and New Drugs Research, and Shaanxi Collaborative Innovation Center of Chinese Medicinal Resources Industrialization, Shaanxi University of Chinese Medicine, Xi'an 712046, China

## Abstract

Ginkgo ketoester tablets (GT) and donepezil were a clinically used combination for the treatment of Alzheimer's disease (AD). The aim of the study was undertaken to investigate the antiamnesic effects of the two drugs alone and in combination through *in vivo* models of the Morris water maze along with *in vitro* antioxidants, acetylcholinesterase (AChE) and butyrylcholinesterase (BuChE). The potential mechanisms were speculated by the activities of acetylcholine (ACh), AChE, superoxide dismutase (SOD), and malondialdehyde (MDA) and the protein expression of brain-derived neurotrophic factor (BDNF) and tyrosine protein kinase B (TrkB). The combination group showed a concentration-dependent inhibition of cholinesterase and antioxidation. As far as its mechanism was concerned, the combination of two drugs exerted excellent effects on oxidative stress, cholinergic pathway damage, and inactivation of the BDNF-TrkB signaling pathway. Additionally, to elucidate the binding mechanism of GT active ingredients into the structure of AChE, the results of molecular docking studies indicated that hydrogen and/or hydrophobic bonds might play an important role in their binding process. Thus, the combination of drugs could treat AD perfectly and further verify the scientific rationality of clinical medication.

## 1. Introduction

As the most common form of dementia among the elderly, Alzheimer's disease (AD) is expected to have rising prevalence with the aging demographics of human society worldwide [1, 2]. The clinical features of AD, including progressive loss of memory, cognitive function, and behavior impairment, make this chronic disease a great threat to human health and quality of life [1, 3]. It was estimated that 35.6 million people lived with dementia worldwide in 2010, with the number of patients expected to double every 20 years [4, 5]. Epidemiological analysis has predicted that the number of people with AD will rise to 1.25 billion by 2050 [4]. As life expectancy is increasing worldwide, the incidence of AD continues to rise significantly, which causes a heavy burden on family and society. Undoubtedly, in-depth research on the pathogenesis and treatment of AD has gained the utmost social and national attention.

AD was thought to be complex and remained elusive, and it was generally believed that it might be related to genetic, biochemical, neuroendocrine, immune, and environmental factors based on aging. In recent years, several hypotheses based on the following factors were proposed to explain the mechanism of AD pathogenesis, such as *β*-amyloid deposition as the core of senile plaques, hyperphosphorylation of tau protein as the main component of neurofibrillary tangles, cholinergic neuron degeneration, or death [6–8]. Additionally, more and more reports indicated that oxidative stress response promoted the synthesis of the A*β* protein by activating positive feedback regulation of A*β* precursor protein *γ*-secretase and *β*-secretase cleavage [6–8].

There are many hypotheses about the pathogenesis of AD, and more and more evidence suggests that oxidative stress plays a key role in the memory of elderly [9–11]. Previous reports indicated that the increased expression of biomarkers associated with oxidative stress such as malondialdehyde (MDA) and reactive oxygen species (ROS) in brain cells and other neural tissue. Brain tissue was particularly susceptible to oxidative stress due to its high consumption of oxygen, low antioxidant capacity, and relatively high content of iron and polyunsaturated fatty acids. Within the brain, these areas were most vulnerable to oxidative stress including the basal forebrain and amygdala. As these regions were important for brain-specific functions such as cognition and memory, damage to these areas could have significant neurological effects. Additionally, several studies reported the presence of elevated DNA, RNA, protein, and lipid oxidation in brains of patients with AD and mild cognitive impairment (MCI). Thus, the development of antioxidants might be useful for improving cognitive function in AD. Recently, more and more attention has been paid on the antioxidants from natural plants to protect the human body from oxidative damage induced by free radicals especially brain tissues [12].

Further, as early as 1982, the destruction of the cholinergic neurotransmitter system was found to be a major factor in age-related central nervous system dysfunction and cognitive loss [13]. This was consistent with the current findings that cholinergic neuron damage was a key pathological change associated with cognitive dysfunction in AD [14]. Acetylcholine (ACh), closely associated with AD, was a neurotransmitter responsible for the cognitive function, learning, and memory. According to the “cholinergic hypothesis,” acetylcholinesterase (AChE), a key enzyme in biological nerve conduction, was mainly associated with the degradation of ACh [15, 16]. In addition, there were some studies on neurotransmitter dysfunction such as dopamine and 5-hydroxytryptamine, but not much as ACh in AD [17]. Therefore, damage of the cholinergic pathways in the brain can bring out cognitive and memory deficits caused by AD.

Numerous studies also showed that the brain-derived neurotrophin factor- (BDNF-) tyrosine protein kinase B (TrkB) signaling pathway played an important role in the progression of AD. Recent research found that increased expression of BDNF mRNA and the activation of TrkB were beneficial for memory consolidation and acquisition [18]. The abnormal expression of BDNF and inactivation of BDNF-TrkB will result in abnormal cognitive function. So some scholars believed that the BDNF-TrkB signaling pathway will become a potential treatment for AD [19]. Recently, increasing evidence suggests that oxidative stress, cholinergic pathway, and BDNF-TrkB signaling pathway participate in the process of AD.

On the basis of various AD pathogenesis, a series of therapeutic drugs were developed, including cholinesterase inhibitors, cholinergic receptor agonists, anti-A*β* therapeutic drugs, calcium ion (Ca^2+^) inhibitors, antioxidant drugs, and anti-free radical drugs, but the effect of treatment was not encouraging especially in brain damage caused by oxidative stress [6, 20]. The understanding of AD in traditional Chinese medicine from the perspective of holistic observation was consistent with the current discovery of AD by Western medicine that AD was a complex systemic disease involving multiple targets. At present, more and more clinical practice has found that the combination of Chinese and Western medicine for the treatment of AD has significant curative effect and the cognitive ability and systemic function of patients are greatly improved with less adverse reactions. Literature reported that *Ginkgo biloba*, *Panax ginseng*, *Rehmannia glutinosa* Libosch, *Epimedium*, *Polygala tenuifolia* Willd, and so on, were usually used to treat AD [21]. Memantine, tacrine, and donepezil were the currently best useful Western medicine.

Among the many combinations of traditional Chinese and Western medicine, the combination of ginkgo ketone ester and donepezil was the most attractive and could reduce the adverse event rate, which was widely used in clinical practice [22–24]. At present, the most potential target for the symptomatic treatment and delay in the development of AD was cholinesterase inhibitors [25]. Donepezil, a selective and reversible inhibitor of AChE, could inhibit AChE activity, enhance cholinergic function, and improve the cognitive function of AD patients [26]. In addition, donepezil was the first-line anti-AD treatment drug recommended by both EFNS and APA guidelines. It has been proven that it was effective and safe in both pathological and clinical studies. *Ginkgo biloba* (*G. biloba*) has been commonly used to mitigate neurodegenerative diseases, intermittent claudication, tinnitus, and many other diseases. Terpene lactones and flavonoids were considered to be the main active components for their beneficial effects. The ginkgo ketoester tablet (GT) is a clinically common Chinese patent drug and is presented with two major active ingredients. Flavonoids from the ginkgo extract exhibited various pharmacological activities including antioxidant, anti-inflammatory, antibacterial, and inhibitory activities of some enzymes [25]. Increasing evidence has shown that flavonoids could inhibit the development of AD-like pathology and reverse deficits in cognition, suggestive of potential improvement in memory and learning in both animals and humans [27–29]. Moreover, it was reported that GT antagonized the neurotoxicity of A*β* [30], improved cerebral circulation and cognition function [31], and possessed a certain neuroprotective effect [32, 33]. In population-based studies, neuropsychiatric symptoms were found in the majority of patients with dementia [34–36], and findings from clinical trials suggest that GT as well as donepezil may improve such symptoms. Experimental research found that GT combined with donepezil was significantly superior to those used alone in improving cognitive function of AD patients [37]. Furthermore, the donepezil adverse effects of cholinergic could be significantly reduced after combination [24]. Additionally, the results of animal experiments also showed that the combination of GT and donepezil could significantly increase the level of ACh compared with donepezil alone, while the use of GT alone did not obviously change the level of ACh [23]. These results indicated that the combination of GT and donepezil possessed synergistic effects in the treatment of AD. However, the mechanism of the synergistic effect between the two drugs was not clear. The objective of the present study was to illuminate the mechanism of the synergistic effect between the two drugs in the treatment of AD based on oxidative stress, cholinergic pathway, and BDNF-TrkB signaling pathway.

## 2. Materials and Methods

### 2.1. Materials and Reagents

Butyrlycholinesterase (BuChE), acetylcholinesterase (AChE, from *electron eel*), acetylcholine iodide (AChEI), butyrylcholine iodide (BuChEI), and 5,5-dithio-bis-nitrobenzoic acid (DTNB) were purchased from Shanghai Yuanye Bio-Technology Co., Ltd. (Shanghai, China). Mouse ACh kit and AChE kit were offered by Yi Fei Xue Biotechnology Co., Ltd. (Nanjing, China). Mouse SOD kit and MDA kit were obtained from Nanjing Jiancheng Bioengineering Institute (Nanjing, China). BDNF and TrkB antibodies were purchased from Nanjing Jin Yibai Biological Technology Co., Ltd. (Nanjing, China). 2,2-Diphenyl-1-picrylhydrazyl (DPPH) was obtained from TCI Chemical Industry Co., Ltd. (Shanghai, China); 2,2′-azino-bis-3-ethylbenzothiazoline-6-sulfonic acid (ABTS) was purchased from Sigma-Aldrich (USA). Ginkgo ketoester tablets were offered by Jiangsu Shenlong Pharmaceutical Co., Ltd. (Yancheng, China) with at least 30% contents of flavonoids and lactones; donepezil (10 mg/piece) was purchased from Eisai China Inc.; scopolamine hydrobromide was obtained from Aladdin Reagents (Shanghai, China); and ultra-pure water was purified by an EPED super purification system (Nanjing, China). Other reagents and chemicals were of analytical grade.

### 2.2. Preparation of Therapeutic Drugs

Scopolamine was dissolved in 0.9% saline. Donepezil and GT were grinded to powder. An appropriate amount of powder was suspended in quantitate 0.005% carboxymethyl cellulose sodium (CMC-Na) salt solution. Animal doses were calculated according to the body surface area conversion factor: human clinical dose × 0.0026/20 × 1000 × multiple of clinical equivalent; donepezil clinical dose was 5 mg and GT clinical dose was 450 mg, and the calculated clinical equivalent of donepezil was 0.65 mg/kg and the GT was 58.5 mg/kg in mice. According to previous reports, the dose of donepezil was 1.0 mg/kg which exhibited good therapeutic effects in the similar modeling method (scopolamine 3 mg/kg) [38]. Additionally, using other modeling methods of Alzheimer's disease, the dose of donepezil was 0.65 mg/kg (clinical equivalent of donepezil) and the Morris water maze results showed significant difference [39, 40].

### 2.3. In Vitro Activity Evaluation

#### 2.3.1. DPPH Radical Scavenging Assay

DPPH is a very stable nitrogen-based free radical and has a UV-vis absorption maximum at 517 nm. The antioxidant activity of the test samples was estimated through DPPH radical scavenging capability according to the method of Brand-Williams et al. with slight modifications [41]. DPPH solution was prepared by dissolving 100.0 mg of DPPH in 100.0 mL of ethanol. A DPPH stock solution was freshly prepared before the tests. The test sample stock solution was prepared in the concentration of 1.0 mg/mL and then diluted into 500, 250, 125, 62.5, 31.05, and 15.625 *μ*g/mL solutions. Then, 100.0 *μ*L DPPH solutions were added to 100.0 *μ*L serial dilutions of the test sample in a 96-well microplate. The reaction mixtures were left for 30 min in dark conditions, and the absorbance was calculated at 517 nm by the EnSpire microplate reader (Perkin-Elmer, United States). All measurements were obtained in triplicate, and the results were recorded as mean ± SD. The radical scavenging activity of the test substance was calculated according to the following formula:

(1) $$\begin{array}{c} \%\text{Radical scavenging}=\left[\frac{\left(A_{0}-{A_{0}}^{\prime }\right)–\left(A_{1}-{A_{1}}^{\prime }\right)}{\left(A_{0}-{A_{0}}^{\prime }\right)}\right]\times 100\%, \end{array}$$

where *A*_0_ is the blank control, *A*_0_′ is the absorbance of the solvent blank and ethanol, *A*_1_ is the absorbance of sample and DPPH, and *A*_1_′ is the absorbance of sample and ethanol. Antiradical DPPH activity is expressed as IC_50_ (mg/mL) which denoted the concentration of the sample required to scavenge 50% of DPPH free radicals.

#### 2.3.2. ABTS Scavenging Assay

The ABTS radical scavenging activity was referred to literature reported, with minor modifications [42]. ABTS and potassium persulfate were dissolved in purified water (7 mM and 2.45 mM, respectively), mixed thoroughly, and stored in the dark at room temperature for 12–16 h to produce free radicals. Then, the ABTS stock solution was diluted with phosphate buffer saline (PBS, pH 7.4) to an absorbance of 0.7 ± 0.02 at 734 nm before usage. The reaction was performed in a 96-well plate, and 200 *μ*L reaction solutions contained 100 *μ*L test samples of different concentrations. The absorbance of ABTS at 734 nm was measured using an EnSpire microplate reader after keeping for 6 min in a dark room. All measurements were obtained in triplicate, and the results were recorded as mean ± SD. The radical scavenging activity of the test substance was calculated according to the following formula:

(2) $$\begin{array}{c} \%\text{Radical scavenging}=\left[\frac{\left(A_{0}-{A_{0}}^{\prime }\right)–\left(A_{1}-{A_{1}}^{\prime }\right)}{\left(A_{0}-{A_{0}}^{\prime }\right)}\right]\times 100\%, \end{array}$$

where *A*_0_ is the absorbance of ABTS and solvent, *A*_0_′ is the absorbance of purified water and solvent, *A*_1_ is the absorbance of the test sample and ABTS, and *A*_1_′ is the absorbance of the test sample minus ABTS.

#### 2.3.3. Anti-Cholinesterase Assays

In this assay, the test samples were performed spectrophotometrically for AChE and BuChE inhibition potential using AChEI and BuChEI as substrates following the method of Ellman et al. with minor changes [16, 43]. The test sample stock solution was prepared in the concentration of 1.0 mg/mL and then diluted into 500, 250, 125, 62.5, 31.05, and 15.625 *μ*g/mL solutions in order to select the appropriate concentration range of IC_50_. Seven different concentrations of test samples were added to the wells of a 96-well microplate containing 10 *μ*L AChE or BuChE (2 U/mL), and then 100 *μ*L phosphate buffer (200 mM, pH 7.7) was added to them. Next, 50 *μ*L DTNB was added in the above solution and incubated for 5 min at 25°C. After incubation, 15 *μ*L of substrates was added and incubated for another 5 min at 25°C. A yellow color developed due to the formation of 5-thio-2-nitrobenzoate anion by the reaction between thiocholines and DTNB was measured at 412 nm. All experiments were repeated three times, and the results were expressed as mean ± SD.

### 2.4. In Vivo Activity Evaluation

#### 2.4.1. Acclimation and Classification of Animals

A total number of 50 ICR mice (18–22 g) in age 5 weeks were supplied by the Comparative Medicine Center of Yangzhou University (Yangzhou, China) with eligibility certification No. SCXK 2017-0001, and they were kept in 22 ± 2°C and 50 ± 5% relative humidity with a 12 h light-dark cycle and allowed free access to water and standard laboratory chow. The experimental protocols were approved by the Animal Experimental Ethical Committee of Nanjing University of Chinese Medicine, and all the procedures were strictly conducted in accordance with ethical principles of animal use and care. The animals were acclimated for one week prior to experiment.

**Figure 1:**
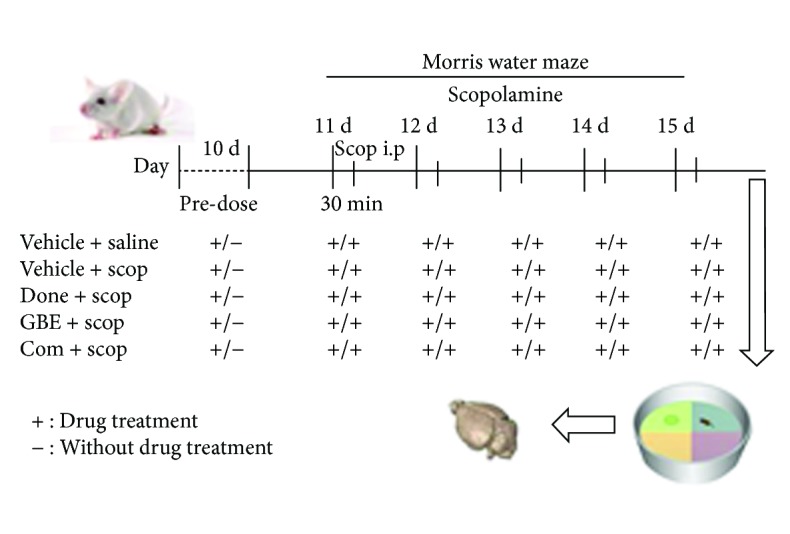
Schematic representation of the experimental design to study the effects of single and combination group drugs on memory impairment.

After 7 days of acclimatization, the mice in age 6 weeks (25–30 g) were randomly divided into five groups (*n* = 10 in each group): (1) control group, which was treated with saline alone; (2) scopolamine group, which was injected with scopolamine alone; (3) donepezil group, which was injected with scopolamine and treated with donepezil (0.65 mg/kg); (4) GT group, which was injected with scopolamine and treated with GT (58.5 mg/kg); and (5) combination group, which was injected with scopolamine and treated with donepezil plus GT (0.65+ 58.5 mg/kg). The timetable of the experiments is shown in Figure 1. For the control group, saline was given, and the other treatment groups were given their respective drugs orally administrated daily for 10 consecutive days using intragastric gavage. From the 11th day onward, scopolamine (3 mg/kg) [44, 45] was injected intraperitoneally to each group except the control group. The mice were orally administrated with drugs 30 min before intraperitoneal injection with scopolamine. After another 30 min, the Morris water maze was carried out for 5 days.

#### 2.4.2. Morris Water Maze

To evaluate the therapeutic effect of the drugs, a 6-day test was conducted for spatial reference learning and memory in a water maze. The water maze consists of a large circular pool filled to a depth of 30 cm with water at 20 ± 2°C. A platform, in the center of one quadrant of the pool, was placed inside the pool 0.5 cm below the surface of water. The Morris water maze test was performed during 11-16 days. The position of the platform was fixed, and the mice, facing the pool wall, were thrown into the water, freely swimming for 60 s. If the mice failed to find the platform within 60 s, the train ended and then the mice were guided to the platform and kept on it for 10 s. The mice entered the water training from 2 different quadrants every day, keeping the position of the platform and surrounding objects constant, and were trained continuously for 4 days. The orientation navigation test was conducted on the 5th day for 60 s, and the time taken for the mice to reach the platform was recorded. Those which could not find the platform were counted as 60 s. On the 6th day, the platform was removed and the space exploration test was conducted. The mice were given a probe trial in which they had 60 s to search for the platform. The latency to reach the platform, the swim distance, and the number of platform location crossings were film-recorded by a camera, mounted above the center of the water maze.

#### 2.4.3. Tissue Preparation

Immediately after the completion of behavioral tests, the animals were sacrificed. The cerebral cortex and hippocampus were rapidly isolated on ice and snap-frozen in liquid nitrogen before transfer to a −80°C refrigerator.

#### 2.4.4. Hematoxylin and Eosin Stain

In each group, three mouse brain tissues were fixed in 4% paraformaldehyde and then embedded in paraffin and coronally sectioned into 4 *μ*m slices for HE stain.

#### 2.4.5. ELISA Kit

The cerebral cortex and hippocampal tissue were dissected from brains and rapidly homogenized with 10 volumes of ice-cold physiological saline, and then centrifuged at 3000 rmp for 15 min at 4°C. The supernatants were collected for further analysis. ACh, AChE, SOD, and MDA activities were measured by commercial available kits. All procedures were carried out according to the manufacturer's protocol using a microplate reader to measure the absorbance at their respective absorption wavelengths.

#### 2.4.6. Western Blot Analysis

The brain tissue was added with physiological saline, homogenized with a homogenizer, and centrifuged at 3000 rpm for 5 min. The supernatant was lysed with RIPA 40 *μ*L for 30 min on ice and centrifuged at 12000 rmp for 30 min at 4°C. The supernatant, which was used to extract the protein sample, was subjected to western blot analyses. Samples after preprocessing were subjected to sodium dodecyl sulfate-polyacrylamide gel electrophoresis (SDS-PAGE) and then transferred to polyvinylidene fluoride (PVPD). Next, the strips were incubated for 1 h with a blocking solution (3% skim milk) and placed on a shaker, at room temperature. The protein bands were visualized by an enhanced chemiluminescence detection system after incubation with primary and secondary antibodies, respectively.

### 2.5. Docking Procedure

**Figure 2:**
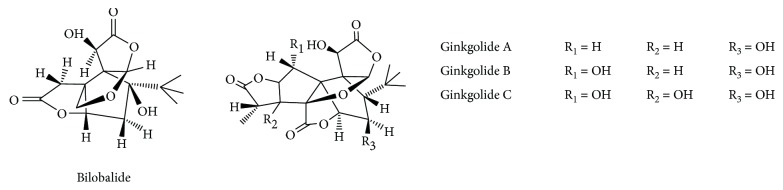
Chemical structure of ginkgolides A, B, and C and bilobalide.

An in silico protein-ligand docking was employed to analyze the binding affinities and modes of terpene lactone (Figure 2) binding to AChE using the AutoDock 4.2 program. The docking studies were performed according to a standard procedure: (1) the crystal structure of AChE (PDB code: 1DX6) was obtained from the RCSB Protein Data Bank; (2) water molecules and unnecessary substructures were deleted; (3) polar hydrogen atoms were added to AChE; (4) Gasteiger charges were calculated for each atom of AChE; (5) an autogrid was run to get grid maps; and (6) final conformations were generated by the Lamarckian genetic algorithm (LGA) by running 100 times. The interaction figures of the compounds binding with AChE were generated with binding energy. (7) The estimated free energy of the compound binding (kcal/mol) and the inhibition constant (Ki) for each compound were calculated using the AutoDock (version 4.2) software tool. The best pose was optimized using Discovery Studio Visualizer 2016 (Accelrys Software Inc.) and PyMol (The PyMOL Molecular Graphics System) programs for the interaction between small molecules and protein, including hydrogen bonding and van der Waals.

### 2.6. Data Analysis

Data obtained from *in vivo* and *in vitro* experiments were recorded as mean ± SD and analyzed with GraphPad Prism (version 5.01) for graphical representation. The statistical results were conducted with Newman-Keuls multiple comparison test in ANOVA, and *P* < 0.05 was regarded significantly different.

## 3. Results and Discussion

### 3.1. The Results of In Vitro Study Results

#### 3.1.1. DPPH and ABTS Scavenging Assay

Table 1 shows the results for the antioxidant properties of donepezil, GT, and combination. To characterize the different mechanisms naturally involved in antioxidant activity, two antioxidant assay methods were applied. According to the results, GT and combination groups showed higher antioxidant activities (DPPH and ABTS methods) than the donepezil group did (*P* < 0.001). The antioxidant activity of different concentrations of GT and the combination against DPPH and ABTS free radicals showed a dose-dependent response, while donepezil had no significant antioxidant activity. In DPPH assay, GT and the combination of two drugs possessed antioxidant activity and the values of IC_50_ were 100.55 ± 1.48 *μ*g/mL and 83.09 ± 1.85 *μ*g/mL, respectively, and they had a significant difference (*P* < 0.001). Similar results were also presented in ABTS assay for the antioxidant properties of donepezil, GT, and combination. GT and the combination groups with the values of IC_50_ were 123.55 ± 1.13 *μ*g/mL and 110.29 ± 1.69 *μ*g/mL, respectively (*P* < 0.001). Additionally, the activity of antioxidant was significantly increased in the combination group (*P* < 0.001), compared with donepezil and GT groups.

Donepezil possessed a weak antioxidant activity from the previous reports. The results of the present study were in accordance with a previous study which showed that donepezil exhibited an insignificant effect on inhibiting DPPH and ABTS radical. The structure of donepezil has N-benzylpiperidine and indanone moieties which showed low antioxidant ability and might explain the above result of donepezil with weak antioxidant activity. Flavonoids were natural products of the benzopyran class, constituting an important group of oxygen heterocycles that possessed high antioxidant ability and were widely distributed in *G. biloba* as secondary metabolites. GT were conventional ginkgo products with at least 24% flavonoids and 6% lactones. Therefore, the strong antioxidant ability was presented in the GT group. The antioxidant activities of donepezil, GT, and combination groups decrease in the following order: combination group > GT group > donepezil group. This might be attributable to the synergistic effect in the combination group with more complex components than those in other groups.

#### 3.1.2. Anti-cholinesterase Activity Assays

Similarly, donepezil, a cholinesterase inhibitor, was the first-line drug for the treatment of AD. In Table 2, it shows the results of AChE and BuChE inhibition by various doses of donepezil, GT, and the combination. Results manifested that donepezil and the combination showed dose-dependently for inhibiting AChE (IC_50_ values of 61.05 ± 1.04 *μ*g/mL and 54.78 ± 0.26 *μ*g/mL, respectively) and BuChE (IC_50_ values of 65.39 ± 0.26 *μ*g/mL and 58.57 ± 0.59 *μ*g/mL, respectively) enzymes. This experimental study found that GT had weak inhibition of cholinesterase, which might be related to the role of its major components, while donepezil and the combination could effectively inhibit cholinesterase. Compared with the combination group, there was a significant difference between the two treatment groups (*P* < 0.001). AChE existed in cholinergic synapse that could degrade ACh, stop the excitatory effects of neurotransmitters on the postsynaptic membrane, and ensure the normal transmission of neural signals in the body. BuChE was a nonspecific enzyme synthesized by the liver and existed everywhere through the human body. It could hydrolyze various types of choline esters. AChE was the main cholinesterase, while BuChE just played a supportive role in the human body. Inhibition of both enzyme activities can achieve the purpose of improving the deficit of cognitive and memory.

From the previous reports, donepezil was currently the most widely prescribed pharmacological agent for the treatment of AD which exhibited selectivity for the inhibition of AChE over BuChE. Stein et al. reported that ginkgo extract (EGb 761) and cholinesterase inhibitors (such as donepezil) presented effective in the treatment of dementia patients [23]. In neuroprotective effects, EGb 761 was reported to elevate brain levels of certain neurotransmitters such as dopamine, noradrenaline, and acetylcholine. Additionally, while EGb 761 alone had no effect, donepezil and the combination of donepezil and EGb 761 increased basal ACh levels by 2- to 3-fold. Concomitantly, significant reductions of AChE were measured in both groups. In this study, the anticholinesterase activities of donepezil, GT, and combination groups decrease in the following order: combination group > donepezil group > GT group. We found that donepezil and GT displayed a slight pharmacological interaction when given together. Adding GT modified the effects of donepezil on the cholinergic system. Therefore, the combination of GT and donepezil might be beneficial in the treatment of AD.

### 3.2. The Results of In Vivo Activity Study

#### 3.2.1. Morris Water Test

Scopolamine, as an inhibitor of the central nervous system, was used widely to generate amnesia in animal models and was able to lead to a transient disruption of memory by increasing the AChE activity [46–48]. As a result, in this study, the neuroprotective and memory-enhancing effects of donepezil, GT, and the combination had been investigated based on an animal model of amnesia. The Morris water maze was often used to study the brain regions associated with spatial learning and memory function evaluation. Therefore, the effect of drug therapy on scopolamine-induced learning and memory deficits was evaluated by the Morris water maze test.

**Figure 3:**
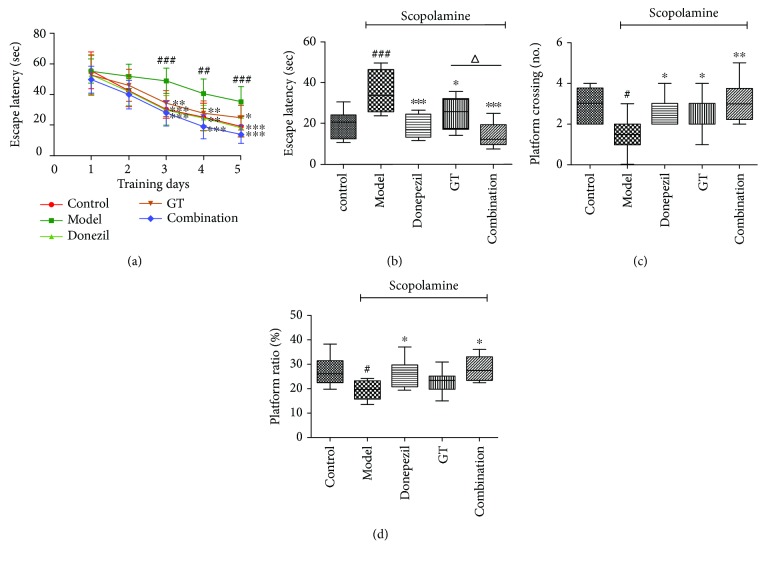
Effects of single and combination group drugs on scopolamine-induced cognitive impairment by the Morris water maze task. Escape latency of each mouse for five consecutive days (a) and escape latency (b) at day 5 of place navigation trial. Number of platform crossing (c) and distance ratio in the target quadrant (d) at day 6 of space exploration. Data are expressed as mean ± SD (*n* = 8 − 10). ^#^ *P* < 0.05 and ^###^ *P* < 0.001 versus control group; ^∗^ *P* < 0.05, ^∗∗^ *P* < 0.01, and ^∗∗∗^ *P* < 0.001 versus model group; ^△^ *P* < 0.05 versus treated groups.

**Figure 4:**
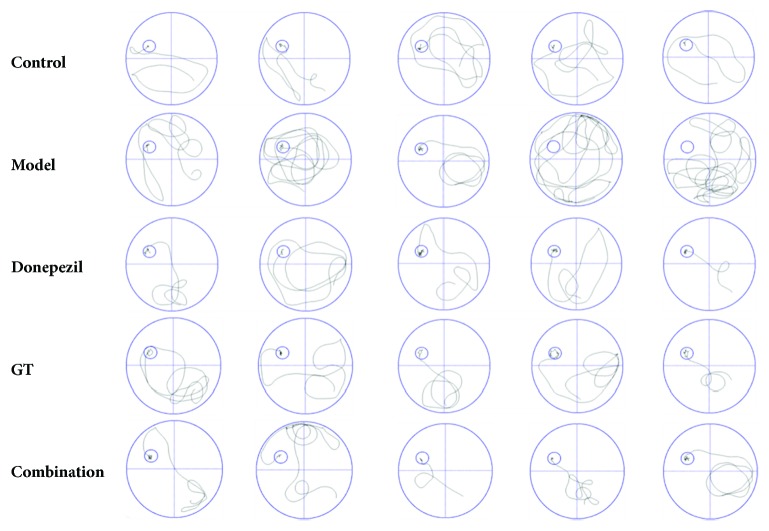
Representative swimming paths. The swimming path of the model group was disordered, while it became clear and concise after administration.

The experimental results could be seen in Figure 3. It was seen that (Figure 3(a)) all groups had no significant difference on the first training day (*P* > 0.05). The scopolamine-induced mice showed longer escape latency than the control group did since the second day and exhibited a marked difference over time (*P* < 0.01). However, the escape latency decreased by administration of donepezil, GT, and the combination of two drugs with the increase in training days. Among these dosing regimens, the combination group had the best efficacy of treatment and had no obvious effect on learning and memory ability when compared to the control group (*P* > 0.05). During the positioning navigation and spatial probe trial, as shown in Figures 3(b) and 3(c), the escape latency and the number of platform crossings were decreased dramatically by the treatment of all dosing, and the combination group was better than the other two groups. This suggested that the combined effect of the two drugs might be better than that of the single drug, especially in the latent period in which it could be seen that the combination group mice found the platform faster than the GT group mice did (*P* < 0.05). In addition, in Figure 3(d), the percentage of the target quadrant (where platform set on) distance showed that the combination and donepezil mice had better memory than those injected with scopolamine (*P* < 0.05). At last, navigation paths at the fifth day of training proved once again that the learning and memory ability of mice was improved after the administration and they could quickly find the platform. As shown in Figure 4, the representative swimming paths of model mice were longer and more chaotic than those of other groups.

#### 3.2.2. Analysis of HE Staining Results

**Figure 5:**
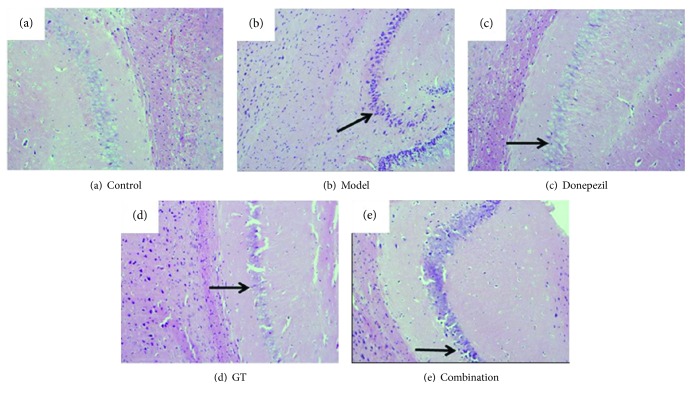
The effects of single and combination groups on neuron morphology in the hippocampus. In the control group (a), the vertebral nerve cell nucleus was round and the nuclear membrane was clear and had nucleoli, while in the scopolamine-induced group (b), the nuclear membrane shrank, the chromatin became dense, and the glial cell increased. In the donepezil group (c), GT group (d), and combination group (e), abnormal cell status returned to normal.

As shown in Figure 5, HE stain showed that the vertebral nerve cell nucleus in the hippocampus of the control group was round, and the nuclear membrane and nucleoli could be observed clearly; while the nuclear membrane of the brain vertebral body shrinks, the chromatin becomes dense and the glial cells increase in the mouse brain, injected by scopolamine. It is worth mentioning that all indicators have improved to some extent after administration, especially the combination group which had the best effect.

#### 3.2.3. Effects of Drug Therapy on ACh Level and AChE Activities

**Figure 6:**
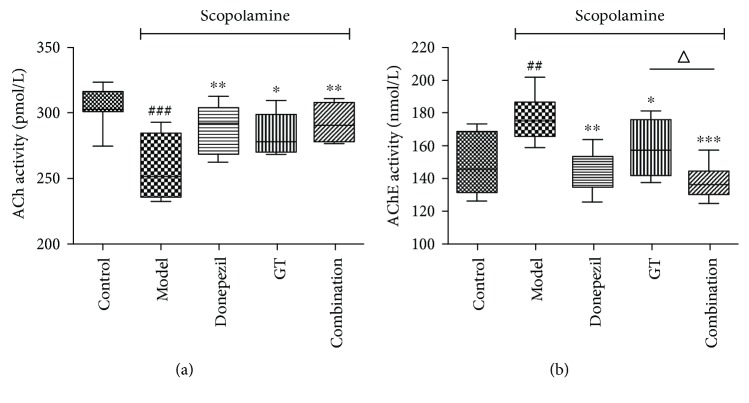
The effects of single and combination groups on ACh and AChE content in the cerebral cortex and hippocampus. After treatment with donepezil (0.65 mg/kg), GBE (58.5 mg/kg), and the compatibility for 16 days, ACh (a) and AChE (b) were determined by biochemical assays separately. Data are expressed as mean ± SD (*n* = 8 − 10). ^##^ *P* < 0.01 and ^###^ *P* < 0.001 versus control group; ^∗^ *P* < 0.05, ^∗∗^ *P* < 0.01, and ^∗∗∗^ *P* < 0.001 versus model group; ^△^ *P* < 0.05 versus treated groups.

In order to elucidate the potential mechanisms of three administration groups in improving cognition deficiency in scopolamine mice, the activities of cholinergic marker enzymes were detected. In Figure 6(a), the results indicated that intraperitoneal injection of scopolamine could significantly decrease the ACh level (*P* < 0.001) when compared with the control group. Surprisingly, preadministration with donepezil, GT, and the combination significantly ameliorated the abnormal changes of the ACh level, in which dosing GT alone had the worst effect (*P* < 0.05) while the other two had similar effects (*P* < 0.01) compared with the model group. In Figure 6(b), the AChE activity was increased by injection of scopolamine in comparison with normal mice (*P* < 0.01), while it was significantly decreased by the treatment with drug groups. Moreover, the combination group exhibited a stronger reversal effect than the other two administration groups and had a significant difference with the GT group (*P* < 0.05).

As we all know, the central cholinergic system played an important role in learning and memory, which was strongly modulated by the neurotransmitter ACh. Therefore, the activity of AChE, a key enzyme hydrolyzing the ACh content in the brain, was considered the definitive marker of central cholinergic function. In this study, we evaluated the mechanism of cognitive dysfunction using scopolamine-induced models. The experiment result showed that three drug groups have the potential to protect the central cholinergic system, and the combination had the best protection. It suggested that the combination group might exert the best effect. This was consistent with the previous literature reports that a combined use could increase the ACh level and significantly reduce the activity of AChE [23]. It indicated that the combination could significantly increase Ach; thus, it might improve the symptom of memory impairment in scopolamine-induced mice.

#### 3.2.4. Effects of Drug Therapy on SOD and MDA Activities

**Figure 7:**
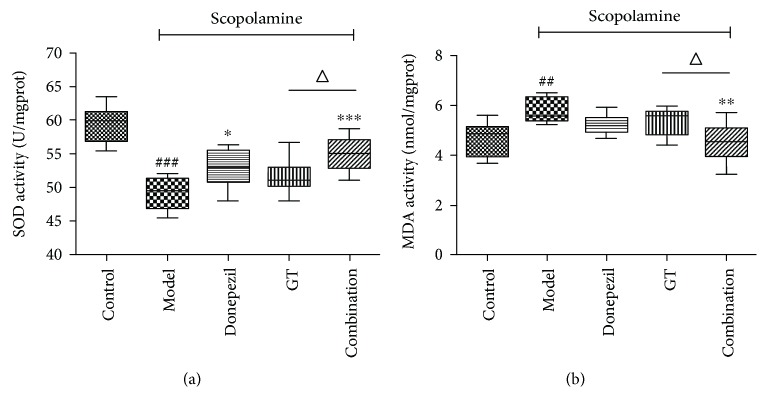
The effects of single and combination groups on SOD and MDA content in the cerebral cortex and hippocampus. After injection of scopolamine, SOD (a) and MDA (b) exerted abnormal expression, while after treatment with donepezil, GT and the combination mice had varying degrees of recovery. Data are expressed as mean ± SD (*n* = 8 − 10). ^##^ *P* < 0.01 and ^###^ *P* < 0.001 versus control group; ^∗^ *P* < 0.05, ^∗∗^ *P* < 0.01, and ^∗∗∗^ *P* < 0.001 versus model group; ^△^ *P* < 0.05 versus treated groups.

The state of brain after scopolamine injection can be determined by the state of oxidative stress. Scopolamine has a robust effect on the brain; that is, the redox state imbalance of the cerebral cortex and hippocampus is aggravated. Therefore, we determined the redox status by measuring the activities of SOD and MDA, respectively.  Figure 7(a) shows that the group with injection of scopolamine was able to decrease the activity of SOD in the brain of mice and had significant difference (*P* < 0.001) compared with normal mice. It was interesting to note that the content of SOD was significantly recovered to varying degrees by drug treatment. In these drug groups, the combination group had the best therapeutic effect and significant difference compared with the GT group (*P* < 0.05). MDA, a marker of lipid peroxidation, represents the levels of oxidative stress [49]. The content of MDA (Figure 7(b)) in the brain of mice was significantly increased after injection of scopolamine (*P* < 0.01), which was consistent with the literature. However, the content of MDA in the brain of the administered mice was dramatically exerted to decrease. In particular, there was a significant difference between the combination group and the GT treatment group (*P* < 0.05). All of this indicated that the combined effects of two drugs were better than those of donepezil and GT alone, which further proved the rationality of the combination.

In the introduction of the free radical aging theory in 1956, the accumulation of free radical damage in cells would lead to aging resulting in memory damage degradation [50]. A study found that the *G. biloba* extract could inhibit the production of free radicals in cardiovascular ischemia, probably due to its SOD-like activity of scavenging hydroxyl radicals [51]. Similarly, the level of MDA, a well-known lipid peroxidation product, had been decreased after the treatment of EGb resulting in an improvement in cognitive function in aged female rates [52]. This may be associated with antioxidant activity of total flavones in *G. biloba* leaves.

#### 3.2.5. Effects of Drug Therapy on BDNF and TrkB Expression in the Cerebral Cortex and Hippocampus

**Figure 8:**
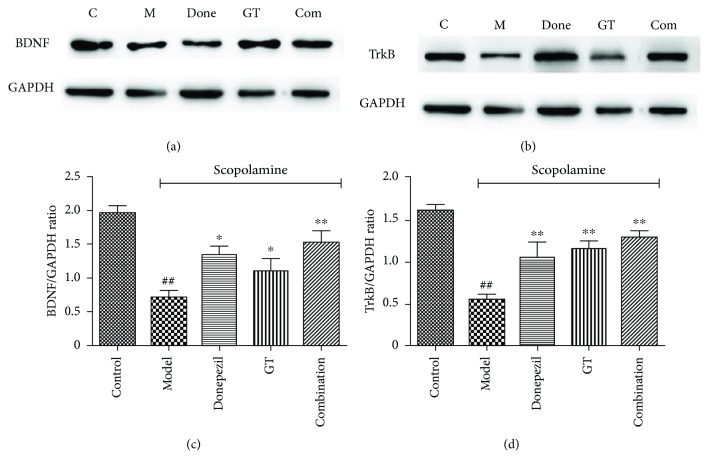
The effects of single and combination groups on BDNF and TrkB expression in the cerebral cortex and hippocampus. Mice were orally preadministrated with donepezil (0.65 mg/kg), GT (58.5 kg/kg), and the combination (0.65+ 58.5 mg/kg) for 10 days and injected with scopolamine (3 mg/kg) to induce amnesia. Western blot analysis of BDNF (a) and TrkB (b) in the cerebral cortex and hippocampus; GAPDH acted as internal control. In addition, bar graphs represent the quantification of the ratio of BDNF/GAPDH (c) and TrkB/GAPDH (d) protein expression. Data are expressed as mean ± SD (*n* = 3). ^##^ *P* < 0.01 and ^###^ *P* < 0.001 versus control group; ^∗^ *P* < 0.05 and ^∗∗^ *P* < 0.01 versus model group.

To verify the crucial molecules in memory formation in the present model, we conducted western blot analyses using tissues in the cerebral cortex and hippocampus. BDNF, a member of the neurotrophin family, played important roles in many developmentally regulated processes via activation of its high-affinity receptor TrkB to modulate cellular function and associated with neurodegenerative disease [49, 53]. Many publications reported that the BDNF-TrkB signaling pathway was involved in AD synaptic damage and was closely related to cognitive dysfunction. Therefore, western blot was used to explore the effect of donepezil, GT, and the combination on the BDNF-TrkB signaling pathway in the cerebral cortex and hippocampus of scopolamine-induced mice. As shown in Figure 8, the activity of the BDNF-TrkB signaling pathway in the brain of model mice was decreased compared with the normal group; the specific performance was that protein levels of BDNF (*P* < 0.01) and TrkB (*P* < 0.001) decreased significantly, implying that injection of scopolamine could inhibit the BDNF-TrkB signaling pathway. Treatment of drugs could prevent scopolamine-induced reduction in BDNF and TrkB expression and make the protein fully express and return to normal levels. The combination group exerted the best effects, although it had no significant difference compared with other administration groups.

BDNF played an important role in regulating the structure and function of neurons and exerted its biological effect through its high-affinity receptor TrkB. The binding of BDNF and TrkB could take part in the regulation of neuronal survival and differentiation, and this signaling pathway played a crucial role in the progression of AD, regulating study and memory [18, 54, 55]. Moreover, this pathway was closely related to the morphology and activity of synapses and played an important role in different stages of synapse development [54]. Neuronal differentiation, synaptic loss, and cognitive dysfunction can occur when BDNF was abnormally expressed or the BDNF-TrkB signaling pathway was inactivated [56, 57]. It could be seen that the role of neuroprotection can be achieved by activating the BDNF-TrkB signaling pathway.

### 3.3. Docking Study

The flavonol glycosides and ginkgolides were the most prevalent in GT. Numerous flavonol glycosides have been identified as derivatives of the aglycones quercetin, kaempferol, and isorhamnetin. From the previous reports, the flavonol glycosides from GT were intracorporeally metabolized as the corresponding aglycones; thus, the aglycones (quercetin, kaempferol, and isorhamnetin) were usually regarded as active compounds when GT were orally administrated in the body. Molecular interaction studies of AChE with the flavonol aglycones (quercetin, kaempferol, and isorhamnetin) from GT were paid more attention and reported in many previous publications. Most reports showed that the AChE inhibitory effect of flavonol aglycones (quercetin, kaempferol, and isorhamnetin) showed good affinity when docked into the AChE-binding site. However, ginkgolides from GT were rarely reported in the previous reports. In this study, molecular docking methods were employed for a comprehensive evaluation of interaction of AChE with ginkgolides (ginkgolides A, B, and C and bilobalide).

**Figure 9:**
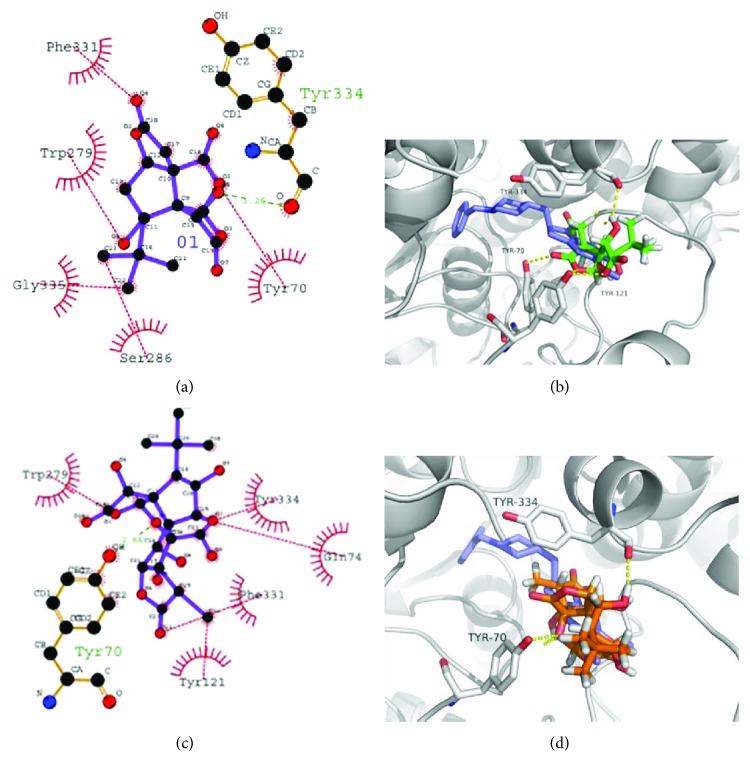
2D ligand interaction diagram of AChE inhibition by bilobalide (a) and ginkgolide C (c). Inhibitory mode of bilobalide (b) and ginkgolide C (d) for the AChE catalytic site. Green and red dashed lines were indicated hydrogen and hydrophobic bonds, respectively. Carbons are in black, nitrogens in blue, and oxygens in red.

**Figure 10:**
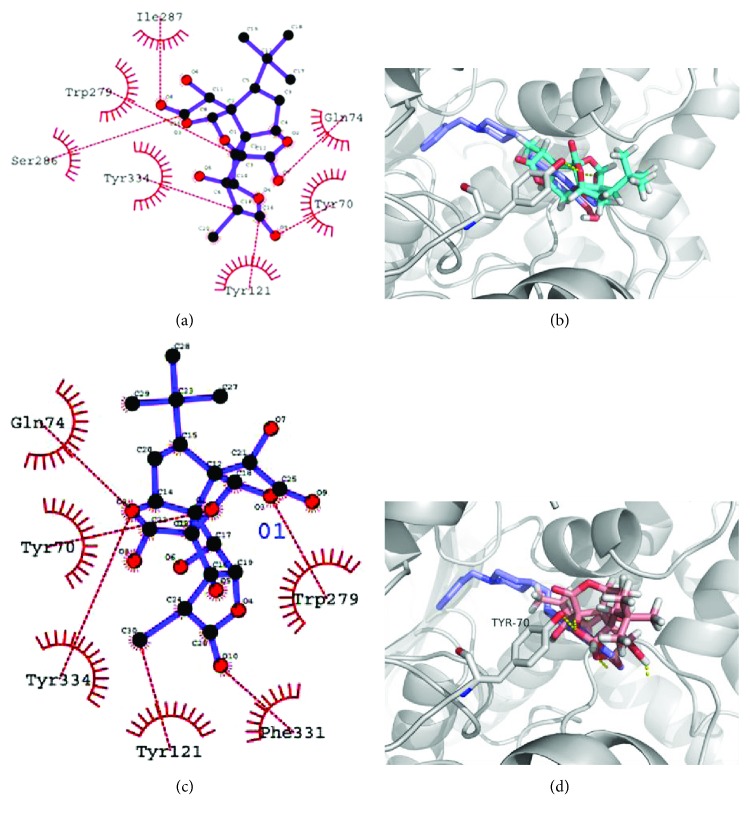
2D ligand interaction diagram of AChE inhibition by ginkgolide A (a) and ginkgolide B (c). Inhibitory mode of ginkgolide A (b) and ginkgolide B (d) for the AChE catalytic site. Green and red dashed lines were indicated hydrogen and hydrophobic bonds, respectively. Carbons are in black, nitrogens in blue, and oxygens in red.

Molecular docking analyses of the series of ginkgolides A, B, C and bilobalide were accomplished into the receptor site of the crystal structures of AChE in order to elucidate the probable mechanism by which the compounds could induce enzyme inhibition activities and to figure out the ligand–protein interaction at the molecular level for establishing structure–activity relationships. From Figures 9(a) and 9(b) and Table 3, bilobalide formed one hydrogen bond with the Tyr334 residue of AChE. Moreover, hydrophobic interactions between bilobalide and Phe 331, Trp 279, Gly 335, Ser 286, and Tyr 70 residues of AchE also seem to be important for binding to the site based on inspection of the AutoDock 4.2 model. The molecular docking model of ginkgolide C is illustrated in Figures 9(c) and 9(d) and Table 3. Ginkgolide C formed a hydrogen bonding interaction with residues like Tyr70. Ginkgolide C also formed five hydrophobic interactions with Phe 331, Trp 279, Gln 279, Tyr 334, and Tyr 121 located in AChE. The illustration of molecular docking for the interactions of ginkgolides A and B with AChE are shown in Figure 10 and Table 3. Ginkgolide A formed 7 hydrophobic interactions with different residues, namely, Ile 287, Trp 279, Ser 286, Tyr 334, Tyr 121, Tyr 70, and Gln 74, while ginkgolide B formed six hydrophobic bonds comprising several residues located in the AChE domain, namely, Gln 74, Tyr 70, Tyr 334, Tyr 121, Phe 331, and Trp 279. No hydrogen bond was observed in ginkgolides A and B with AChE. Additionally, the low content of other ginkgolides (such as ginkgolides J, P, and Q) and AChE was also analyzed by molecular docking studies, and the results indicated that both hydrogen and hydrophobic interactions were observed in ginkgolides J, P, and Q. The molecular docking model of ginkgolides J, P and Q are illustrated in Figure S1 and Table S1 from Supporting Information. Thus, from the present molecular docking studies, hydrogen and/or hydrophobic bonds might play an important role in the binding process of AChE and ginkgolides. From the previous reports, many flavonols and ginkgolides from *G. biloba* could potentially form hydrogen bonding and/or hydrophobic interactions with the AChE active sites [58, 59]. Thus, the synergistic antiamnesic effects between GT and donepezil observed in this study may be due to cooperative synergism of ginkgolides, flavonoids, and donepezil.

## 4. Conclusion

In conclusion, in *in vitro* experiments, GT and donepezil showed their respective antioxidant and inhibitory effects on cholinesterase, and there was a significant difference between combined and single use. An *in vivo* study showed that injection of scopolamine could impair the ability of learning and memory which is accompanied with BDNF and TrkB decline and abnormal expression of ACh, AChE, SOD, and MDA. However, after administration, scopolamine-induced oxidative stress in the brain was inhibited and the BDNF-TrkB signaling pathway was upregulated. To elucidate the binding mechanism of GT active ingredients into the structure of AChE, molecular docking studies were performed and the results indicated that hydrogen and/or hydrophobic bonds might play an important role in the binding process. In summary, GT and donepezil exerted significant synergistic antiamnesic effects by exerting antioxidant and cholinesterase activities and activating the BDNF-TrkB signaling pathway. Therefore, the combination of GT and donepezil could serve as a potential candidate to help treat AD.

## Figures and Tables

**Table 1:** Percent DPPH and ABTS free radical scavenging activity of donepezil, GT, and combination.

Sample	Concentration (*μ*g/mL)	% DPPH scavenging Mean ± SD	IC_50_ (*μ*g/mL)	% ABTS scavenging Mean ± SD	IC_50_ (*μ*g/mL)
Donepezil	1000	22.57 ± 0.59	(−)	13.47 ± 0.16	(−)
	500	16.51 ± 0.91		10.48 ± 0.39	
	250	12.97 ± 0.71		9.50 ± 0.33	
	125	12.43 ± 0.98		6.46 ± 0.43	
	62.5	10.25 ± 0.28		4.48 ± 0.28	
	31.25	8.23 ± 0.51		2.85 ± 0.12	
	15.63	6.91 ± 0.02		1.66 ± 0.09	

GT	1000	91.90 ± 0.42	100.55 ± 1.48	95.77 ± 0.51	123.55 ± 1.13
	500	81.29 ± 0.41		88.10 ± 0.92	
	250	68.35 ± 0.21		77.20 ± 0.96	
	125	55.41 ± 0.28		46.91 ± 0.72	
	62.5	41.53 ± 0.56		26.53 ± 0.33	
	31.25	22.62 ± 0.26		12.96 ± 0.90	
	15.63	14.83 ± 0.46		4.13 ± 0.86	

Combination	1000	94.24 ± 0.66	83.09±1.85^∗∗∗^	99.60 ± 0.18	110.29±1.69^∗∗∗^
	500	84.11 ± 0.23		95.46 ± 0.59	
	250	72.30 ± 0.37		79.13 ± 0.54	
	125	59.48 ± 0.46		48.03 ± 0.81	
	62.5	42.46 ± 0.51		22.49 ± 0.74	
	31.25	29.52 ± 0.77		15.51 ± 0.91	
	15.63	16.58 ± 1.04		7.22 ± 0.18	

Note: Data were given as mean ± SD (*n* = 3).

**Table 2:** Anticholinesterase activity assay results.

Sample	Concentration (*μ*g/mL)	% AChEI Mean ± SD	IC_50_ (*μ*g/mL)	% BuChEI Mean ± SD	IC_50_ (*μ*g/mL)
Donepezil	1000	91.95 ± 0.37	61.05 ± 1.04	91.61 ± 0.28	65.39 ± 0.26
	500	85.29 ± 0.30		85.11 ± 0.72	
	250	76.89 ± 0.34		76.45 ± 0.26	
	125	64.90 ± 0.21		62.80 ± 0.47	
	62.5	50.53 ± 0.73		50.77 ± 0.40	
	31.25	35.26 ± 0.50		37.39 ± 0.28	
	15.63	24.57 ± 0.26		19.02 ± 0.67	

GT	1000	22.79 ± 0.71	(−)	22.41 ± 0.47	(−)
	500	20.36 ± 0.29		20.77 ± 0.71	
	250	18.87 ± 0.58		18.13 ± 0.33	
	125	14.32 ± 0.49		14.89 ± 0.67	
	62.5	11.07 ± 0.49		10.51 ± 0.36	
	31.25	7.45 ± 0.46		7.17 ± 0.86	
	15.63	4.75 ± 0.78		4.44 ± 0.32	

Combination	1000	94.06 ± 0.97	54.78±0.26^∗∗∗^	91.38 ± 0.39	58.57±0.59^∗∗∗^
	500	87.12 ± 0.47		83.07 ± 0.53	
	250	78.89 ± 0.07		73.94 ± 0.35	
	125	67.89 ± 0.67		69.05 ± 0.90	
	62.5	52.76 ± 0.70		54.93 ± 0.95	
	31.25	38.14 ± 0.49		39.19 ± 0.07	
	15.63	27.81 ± 0.62		20.67 ± 0.65	

Note: Data were given as mean ± SD (*n* = 3).

**Table 3:** Docking scores and interactions of compounds (bilobalide and ginkgolides A, B, and C) with AChE.

Compounds	Glide score (kcal/mol)	No. of H-bonds	H-bond interacting residues	Van der Waals interacting residues
Bilobalide	−6.6	1	Tyr334	Phe331, Trp279, Gly335, Ser286, Tyr70
Ginkgolide A	−6.3	0	None	Ile287, Trp279, Ser286, Tyr334, Tyr121, Tyr70, Gln74
Ginkgolide B	−6.0	0	None	Gln74, Tyr70, Tyr334, Tyr121, Phe331, Trp279
Ginkgolide C	−5.8	1	Tyr70	Trp279, Tyr334, Gln74, Phe331, Tyr121

## Data Availability

The data used or analysed during the current study are available from the corresponding author on reasonable request.

## Supplementary Materials

Supplementary Materials The result of the docking study of ginkgolides J, P, and Q. Docking scores and interactions of ginkgolides J, P, and Q with AchE are shown in Table S1. A 2D ligand interaction diagram of AChE inhibition by ginkgolide J (a), ginkgolide P (b), and ginkgolide Q (c) is shown in Figure S1.
