# Supplementary material for: Studies of the Anti-amnesic Effects and Mechanisms of Single and Combined Use of Donepezil and Ginkgo Ketoester Tablet on Scopolamine-Induced Memory Impairment in Mice
Source: Oxid Med Cell Longev. 2019 Feb 18;2019:8636835. doi: 10.1155/2019/8636835 (PMC6398023; doi:10.1155/2019/8636835)
Supplement: Supplementary Materials — The result of the docking study of ginkgolides J, P, and Q. Docking scores and interactions of ginkgolides J, P, and Q with AchE are shown in Table S1. A 2D ligand interaction diagram of AChE inhibition by ginkgolide J (a), ginkgolide P (b), and ginkgolide Q (c) is shown in Figure S1. [file 8636835.f1.pdf]

Supporting Information

Docking Study of Ginkgolide J, P and Q From Figure S1A and Table S1, ginkgolide J established H-bonding with amino acid residues Tyr70 and hydrophobic interactions interactions with Ile287, Phe331, Tyr334, Phe290, Gly335, Ser286, Trp279, Gln74 and Tyr121 inside AChE. The illustration of molecular docking for the interactions of ginkgolide P and AChE was shown in Figure S1B and Table S1. Ginkgolide P formed 4 hydrophobic interactions with different residues namely Gln74, Tyr121, Tyr334 and Trp279. Moreover, a hydrogen bonding interaction with residue like Tyr70 was also observed. The molecular docking model of ginkgolide C is illustrated in Figure 9C and Table S1. Ginkgolide Q formed 2 hydrogen bonding interactions with residues like Tyr70 and Tyr121. Ginkgolide Q also formed five hydrophobic interactions with Tyr334, Phe290, Gly335, Trp279 and Gln74 located in AChE.

TABLE S1: Docking scores and interactions of compounds (Ginkgolide J, P and Q) with AChE.

Compounds	Glide Score (kcal/mol)	No of H-bonds	H-bonds interacting residues	Van der Waals interacting residues
Ginkgolide_J	-6.1	1	Tyr70	Ile287, Phe331, Tyr334, Phe290, Gly335, Ser286, Trp279, Gln74, Tyr121
Ginkgolide_P	-6.5	1	Tyr70	Gln74, Tyr121, Tyr334, Trp279
Ginkgolide_Q	-6.2	2	Tyr70, Tyr121	Tyr334, Phe290, Gly335, Trp279, Gln74

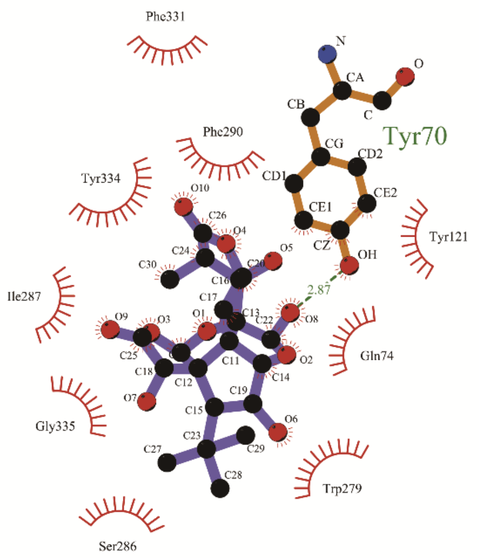

(A)

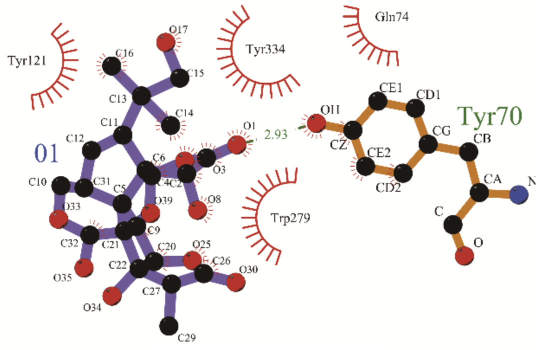

(B)

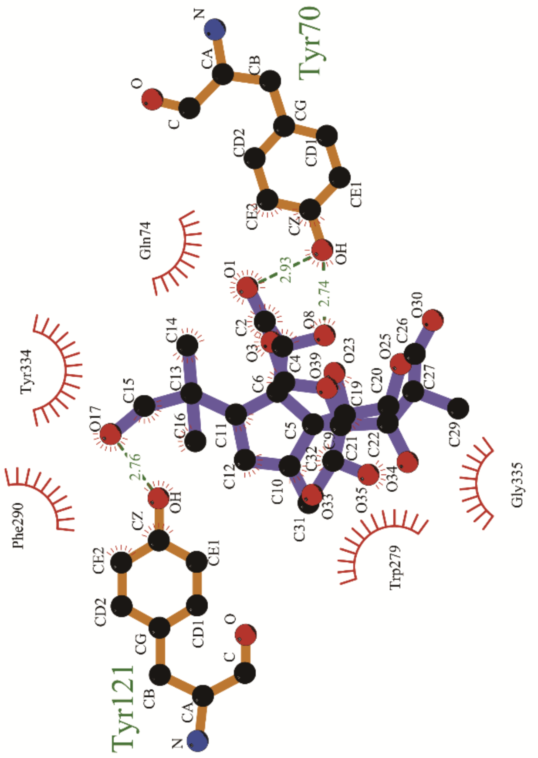

(C)

FIGURE S1: 2D ligand interaction diagram of AChE inhibition by ginkgolide J (A), ginkgolide P (B) and ginkgolide Q (C). Green and red dashed lines were indicated hydrogen and hydrophobic bonds, respectively. Carbons are in black, nitrogens in blue and oxygens in red.
